# Feasibility of a computer-assisted social network motivational interviewing intervention to reduce substance use and increase supportive connections among emerging adults transitioning from homelessness to housing

**DOI:** 10.1186/s13722-022-00307-4

**Published:** 2022-05-03

**Authors:** David P. Kennedy, Karen Chan Osilla, Joan S. Tucker

**Affiliations:** 1 RAND Corporation, 1776 Main Street, PO Box 2138, Santa Monica, CA 90407-2138 USA; 2 Department of Psychiatry and Behavioral Sciences, Stanford University School of Medicine, 401 Quarry Road, Palo Alto, CA 94305 USA

**Keywords:** Social network intervention, Data visualization, Alcohol and other drug use, Homelessness, Transitional age youth, Housing first, Motivational interviewing, EgoWeb 2.0

## Abstract

**Background:**

Social networks may play positive and negative roles in the lives of young adults experiencing homelessness (YEH) who are transitioning into housing. Social networks can influence their alcohol and/or other drug (AOD) use, as well as provide immediate and long-term support necessary for a successful transition.

**Methods:**

We adapted a four-session computer-assisted motivational interviewing social network intervention (MI-SNI) for YEH transitioning into housing. We iteratively adapted and beta tested the intervention for delivery by case managers at an organization that provides supportive housing to YEH. We conducted a focus group with agency staff (n = 6), role-play exercises with case managers (n = 3), and semi-structured interviews with residents (n = 6). Interview data were thematically analyzed with open coding. This study presents the first adaptation of an innovative social network-based motivational intervention to reduce AOD use and increase stable, prosocial supportive connections via visualizations of the structure and composition of the individual’s social network.

**Results:**

Participants rated sessions as “moderately” to “very” helpful and “good” quality on average. Participants agreed that the sessions were helpful, understandable, and satisfying and would work for new residents. Themes emerged in four broad categories: (1) Acceptability, (2) Positive benefits, (3) Visualization reactions, and (4) MI-SNI interface reactions. For the acceptability category, three sub-themes emerged: (1) *understandability*, (2) *enjoyability and ease of use*, (3) and *barriers to acceptability*. Five sub-themes emerged about the intervention’s likelihood to trigger positive benefits: (1) *learning/new insights*, (2) *enhancement to motivation to change*, (3) *making AOD changes*, (4) *building social support*, and (5) the intervention’s *usefulness to some but not others*. Five sub-themes also emerged from comments about the social network visualizations: (1) *general positive comments*, (2) *understandability*, (3) *new insights*, (4) *triggering changes in social support*, and (5) *triggering changes in AOD use*. When discussing the MI-SNI intervention interface beyond the visualizations, discussions emerged in three thematic categories: (1) discussion of *name listing*, (2) discussion of *screen wording*, and (3) the use of a *computer interface* to deliver the intervention.

**Conclusions:**

Results suggest that the MI-SNI is acceptable to YEH and can be feasibly delivered by case managers during case management sessions.

*Trial registration* ClinicalTrials.gov Identifier: NCT04637815. Registered November 10, 2020

## Background

### Substance use disorders among youth experiencing homelessness

Substance use disorders (SUDs) are common among youth experiencing homelessness (YEH). In a study of 198 YEH recruited from street and service sites, 69% met DSM-IV criteria for at least one past year SUD [1]. Another study of 185 YEH recruited from a drop-in center and known by staff to use alcohol and/or drugs found that 80% and 85% met criteria on the MINI International Neuropsychiatric Interview [2] for past year alcohol and drug use disorders, respectively [3]. In a sample of 273 YEH randomly sampled from street sites, 30% screened positive on the GAIN-SS [4] for SUD based on past month use [5]. Having a substance use problem is not only a risk factor for becoming homeless, but is a significant barrier to exiting homelessness [6] and may be a critical factor in the transition to stable housing.

### The role of social networks

Social networks are naturally occurring groups of people that can influence an individual’s behavior in both positive and negative ways through various mechanisms, such as social comparison, social sanctions and rewards, flows of information, support and resources, stress reduction, and socialization [7–9]. The networks of YEH, while diverse, include a high proportion of substance users [10, 11], which is associated with heavier alcohol and drug use [11, 12]. YEH tend to have few stable support providers in their network [13–15] and are more likely to engage in substance use with network members who provide support or occupy other influential social roles [16]. Given the important role of networks in the substance use behaviors of YEH, social network interventions may be a promising approach for supporting youth in their efforts to reduce their substance use while increasing and strengthening stable, pro-social supportive connections. This may help youth achieve other key goals (e.g., finishing education, gaining employment) and aid their successful transition out of homelessness [17].

### Social network interventions

Because of the association between social networks, health and health behaviors [9, 18], health interventions have increasingly incorporated social relationships into interventions [19–23]. Behavior change interventions informed by social network analysis (SNA) have addressed alcohol and/or other drug (AOD) use in a variety of populations [23], including adults in housing programs [24, 25], and can potentially address AOD use among YEH transitioning into housing. Most network intervention approaches are informed by diffusion of innovation theory [26] and aim to affect behavior change throughout a well-defined, clearly bounded, and static network (e.g. school friends). Although YEH form ties with other YEH and the extended network resulting from these connections has ramifications for risk behaviors [27], YEH are also at the center of their own personal networks which consist of a diverse constellation of types of ties (family, housed peers, non-family adult mentors) that influence AOD use and social support in diverse ways [10, 12–15, 17, 28]. The personal networks of YEH transitioning into housing are unlikely to remain static, as they experience increased exposure to new pro-social connections as well as new AOD using peers and other residents of supportive housing programs that do not require residents to abstain from AOD use [29–32].

The “alteration” style of network intervention [22] is perhaps the most relevant network intervention type for assisting YEH as they transition into housing. This approach is used to trigger network changes such as adding or subtracting members of a network or connections among network members, or modifying relationships among network members. Alteration network intervention approaches have been developed to target changes to personal (egocentric) networks [23, 25, 33–35] by using techniques to measure and visualize the immediate social environment of a collection of independently sampled individuals [36–38]. This approach is appropriate for YEH transitioning into housing because each YEH is at the center of an evolving group of interconnected people who play a variety of roles in assisting or hampering this transition.

### A motivational interviewing social network intervention

To the best of our knowledge, our team has developed the only social network intervention that uses personal network visualizations to encourage healthy behavior change through alteration of both the compositional and structural characteristics of personal networks [24, 25, 39]. This computer-assisted intervention involves conducting four sessions with a client during which a facilitator: (a) collects information on the composition and structure of the client’s personal network; (b) shows the client visualizations of their social network immediately afterwards (e.g., the connections between network members, highlighting those who influence the client’s substance use); and (c) helps the client identify aspects of their behavior (e.g., drinking less) or networks (e.g., how much they socialize with certain people) that they want to change between now and the next session. The intervention is delivered by a facilitator using a Motivational Interview (MI) conversational style [40], which is non-judgmental and non-confrontational [40]. This style is important for YEH who often report negative experiences with adults in “helping” roles (e.g., police, case managers, therapists, foster parents) [41, 42] and reluctance to engage in services when they perceive staff to be judgmental [43]. In a pilot randomized controlled trial with adults experiencing homelessness who recently entered supportive housing and reported past year problematic substance use, individuals who received this motivational social network intervention (MI-SNI) had a greater reduction in the proportion of their network members who negatively influenced their alcohol or other drug use (e.g., drinking partners) and, for network members retained in the network across waves, lower exposure to substance use risk with these network members [44]. Further, individuals who received the MI-SNI had greater increases in their readiness to change substance use and substance abstinence self-efficacy, and greater reductions in alcohol use, compared to those who did not receive the MI-SNI [25].

### The present study

We extend our prior work with the MI-SNI by adapting it for YEH (18–25) who have experienced homelessness and recently transitioned into supportive housing. Similar to the approach we used for adults experiencing homelessness who recently entered supportive housing, this intervention is computer- or tablet-assisted so that a case manager can collect personal network information from the resident, show visualizations of their social network immediately afterwards, and discuss potential areas of change using MI. The MI-SNI adapted for youth focuses on both reducing their substance use and increasing/strengthening their stable, prosocial supportive connections that can help them remain stably housed and achieve the goals that they set for themselves. The current paper describes the iterative adaptation of the MI-SNI with residents of a supportive housing program for transition-aged youth experiencing homelessness.

### Intervention conceptual framework

The intervention is delivered using MI and is grounded in complex systems and social capital theories [40, 45–47] and targets improvements in network characteristics including increased contact with supportive network members and decreased contact with those who use AOD. Complex systems and social capital theories emphasize the importance of examining the social network and its influence on the individual’s behaviors [48–50] because this network system can influence the individual positively or negatively [51]. These theories also suggest that changes made in one area of the system may influence the system [50].

Finally, consistent with MI and as explained by the self-determination theory, the intervention emphasizes individual autonomy and innate capacity for growth and change [45, 47]. Through building on the individual’s self-efficacy, the intervention aims to increase the individual’s confidence in their ability to change their behavior [52]. Together, the intervention aims to empower the resident to change their social environment, which we hope will lead tonetwork changing and AOD-related behaviors.

## Materials and methods

### Intervention

We adapted the existing MI-SNI used in our prior study with adults entering supportive housing [24, 25, 39]. We retained the structure and format of the original intervention where facilitators meet with the residents every two weeks for a total of four sessions. In each session, the facilitator interviews the resident with a series of open and closed-ended questions about 15 people in their network they interacted with in the past two weeks and how often these members connected with each other  (network structure). Network composition questions include whether each member was likely to use AOD in the next two weeks,  whether the resident was likely to use AOD with each member, and whether the resident would go to the network member for support in reaching their goals. After the interview, the facilitator and resident review three social network visualizations that are automatically and immediately populated from the preceding interview and the facilitator uses MI to discuss each visualization (e.g., what do you notice about this picture? How does [member1] affect your AOD use?).

**Fig. 1:**
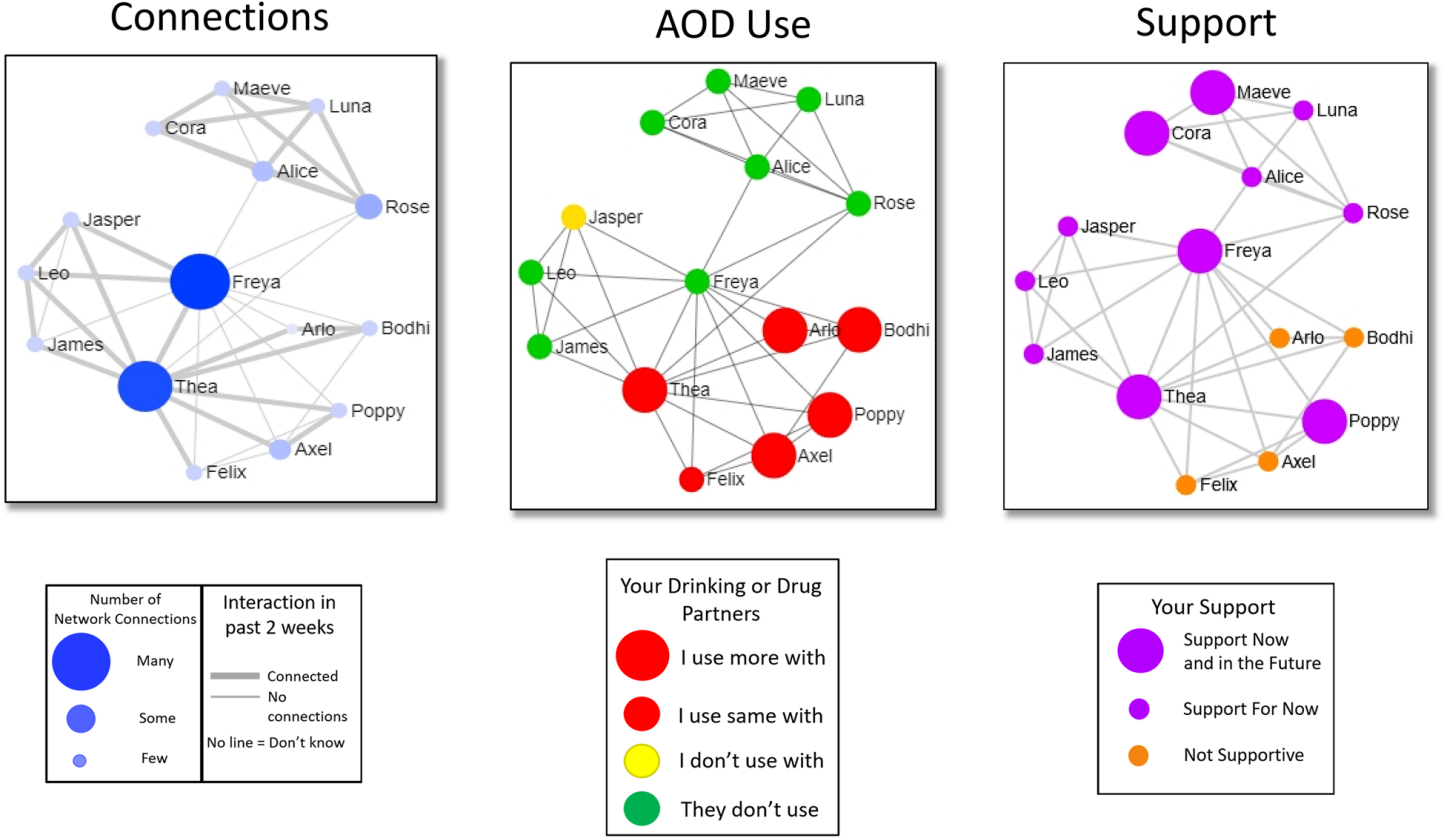
Example figures from hypothetical MI-SNI session. Network contacts are represented by circles (graph “nodes”) and lines between nodes represent network contacts who interacted with each other in the past 2 weeks. The layout of the nodes, generated with the Fruchterman–Reingold force-directed placement algorithm highlights structural characteristics of the network, such as isolates (completely disconnected nodes) and components (a set of nodes tied together but disconnected from other nodes). The structural layout is consistent across the three diagrams. The “Connections” figure on the left (a) uses node color, node size, and line thickness to highlight other characteristics of the network structure, including the centrality of network actors (depicted by larger and darker nodes) and stronger relationship ties between actors (highlighted with thicker lines). The other figures use node size and color to highlight network composition. The AOD use figure in the middle highlights drinking and drug use partners with size and color ranging from small green nodes (no AOD use by network member) to large red nodes (participant uses with more than usual). The “Support” figure on the right depicts network members with size and color (large purple = supportive now and in the future, small purple = supportive now only, small orange = not supportive)

The three visualization types are identical across the four sessions, but each session’s visualizations change depending on the resident’s responses to the network interview, which covers the time since the previous session. For example, it could include new network members and show different interactional patterns (e.g., more contact with prosocial members, less contact with those who use AOD compared to an earlier session). Figure 1 depicts examples of the three visualizations for a hypothetical intervention participant. Each figure presents a visualization of the same network data but highlights different network characteristics.

Network members are represented as circles (nodes) and the relationship between network members is represented by lines connecting nodes. The positioning of the nodes in the graph is a result of a “spring embedding” visualization algorithm [53], which places people who know each other and have similar ties to other network members close together and places people who do not have these qualities further apart. The visualization draws attention to structural features of the network such as isolates (completely disconnected nodes) and components (a set of nodes tied together, but disconnected from other nodes).

The first visualization shows a picture of the resident’s network of 15 members and how members are connected with each other. Line thickness represents frequency of recent contact and node size highlights the number of connections a particular network member has with other members of the network. The second visualization presents the same node positioning as the first visualization but depicts nodes in different colors and sizes based on whether each member used substances and how likely the resident reported using substances with each in the future (e.g. small red = likely to use regular amount or less, large red = likely to use more than regular amount). Finally, the third visualization depicts the resident’s social support by illustrating network members who were not supportive, supportive now, or supportive both now and in the future*.* Network members are classified on their support in reference to helping the resident achieve the top two goals they have for themselves for the next year. The two goals are selected from a list of possible goals, such as getting a job, finishing school, reducing their AOD use, improving physical and/or mental health, etc.

### Setting

This study was conducted in collaboration with an agency that provides supportive housing to YEH in Los Angeles County. This agency provides a comprehensive continuum of care to YEH that includes free emergency resources such as food and clothing in combination with case management, health, vocational, educational, therapeutic, and housing services. Housing is provided without a requirement of AOD abstinence for new residents.

### Participants and procedures

We conducted a four-step process to develop and evaluate the acceptability of the intervention for residents and the feasibility of case managers delivering the intervention directly to their clients. We revised the intervention iteratively between each step and obtained feedback on successive versions of the intervention. All participating staff and residents provided consent to participate, and procedures were approved by the study’s Institutional Review Board.

#### Step 1: Staff focus group

The goal was to obtain initial feedback on the MI-SNI tool, including how residents may respond to the intervention and any wording or interface changes that may improve acceptability, as well as discussion of the logistics of the intervention process, such as recruitment and incentives. Participants were seven residential support staff (2 men, 5 women; 1 White, 1 African American, 5 Latinx) who were case managers, program managers, or administrators identified by the organization’s Resident Services Director for their diverse experience in assisting YEH entering supportive housing. Two members of the research team role-played an MI-SNI session using the initial prototype of the tool that included screen shots of the MI-SNI interface and visualizations. Focus group participants were asked to provide feedback on the screen shots, such as whether the wording or visualizations were difficult to understand or may present problems if used in an intervention session with a resident. We also requested their feedback on the language, structure, and presentation. The discussion was audio recorded and later professionally transcribed. Notes taken by the research project team during the focus group were reviewed along with the focus group transcript for any comments related to suggested changes to the MI-SNI interface or the proposed intervention approach. Research team discussions led to decisions about modifications to the interface, intervention, beta test and pilot test procedures.

#### Step 2: Beta tests with residents, conducted by the research team

Case managers identified four residents (2 Latinx women, 1 White woman, 1 Latinx man) who had experienced substance use problems when they initially entered housing and had resided at the housing site for at least several months. Residents that agreed to participate completed a consent-to-contact form and were scheduled to complete an in-person session. A member of the research team conducted the MI-SNI session with each resident in a private office space in a housing facility. Residents were asked to respond to questions during the MI-SNI session based on their experiences in their first months at the housing facility. Residents completed a semi-structured debriefing session in which they were interviewed by a second research team member about their reactions to the session. These debriefings were similar to the approach of previous feasibility studies of the MI-SNI [40] and other intervention feasibility studies [55–57]. First, residents completed a short survey to obtain their demographic information and ratings of the overall quality (1 = poor to 4 = excellent) and overall helpfulness (0 = not at all to 4 = extremely) of the session. In addition, participants were asked to rate the session in terms of being helpful, understandable, and satisfying (1 = strongly disagree to 5 = strongly agree). The same scale was used by participants to rate how much they agreed/disagreed with eight statements about how well the sessions would work for new residents (e.g. “I would recommend this type of session to another resident”, “The information in the session will cause new residents to change their social networks”). Next, research staff led a discussion with residents who were asked to describe their overall reactions, what they liked best and least about the sessions, their reactions to seeing the network diagrams, what they learned from the session, and what about the session should be changed. Participants were also asked to discuss whether they thought that the network diagrams would help new residents to make changes in their substance use and social networks and if they would recommend the session to a new resident. All interviews were audio recorded and professionally transcribed. Participants were paid $25 for their participation. These sessions lasted between 45 and 60 min.

#### Step 3: Case manager role play

Three case managers (2 females, 1 male; 2 Latinx, 1 African American) from the focus group participated in role play exercises in the offices where they typically conduct their case management sessions with residents. The goal of this exercise was to introduce the MI-SNI interface and visualizations without having to read text on screens and think of probing questions during the discussion of visualizations. Another goal was to assess the feasibility of case managers using the office setting and equipment that they typically used to conduct their case management sessions for the MI-SNI. Case managers first observed two members of the research team conduct a role play of a full session between a case manager and a resident using the MI-SNI tool. While observing the session, the case managers followed along, entering responses into the MI-SNI tool using the office computers to log into the MI-SNI, which was hosted on a project specific virtual machine established by the research team. After observing the role play session and having the opportunity to ask questions about using the tool, case mangers took turns conducting role play interviews with each other. For each role play session, one case manager role-played a resident, while the other conducted the session hands-on using the MI-SNI tool with the research team observing. After this exercise, case mangers provided additional feedback to the research team about what they noticed while using the tool and asking questions. The feedback was generated by the research team asking broad open-ended questions (e.g. “What did you think?”). The open conversation was audio recorded and transcribed. The research team reviewed and discussed this transcript along with notes taken during the sessions to identify changes in the interface or session procedures.

#### Step 4: Beta tests with residents, conducted by case managers

These beta tests involved two new residents (1 female, 1 gender not reported; 1 White, 1 Pacific Islander), but used the same recruitment and consent procedures. The beta tests were facilitated by case managers using the same offices and office computers that are used for their usual case management sessions. These sessions were observed by a member of the research team. After the session, a member of the research team conducted a debriefing session with each beta test resident following the same procedures and interview protocol as described in Step 2. After integrating this second round of beta test feedback, the research team determined that the MI-SNI tool did not need further refinement.

### Analyses

#### Qualitative analyses

Beta test discussion transcripts were imported into analysis software Dedoose [54]. The qualitative procedures and analyses for the staff focus group and resident interview follow procedures for conducting beta test interviews of MI-based intervention tools with adults [39, 55–61], as well as other exploratory interviews with people experiencing homelessness in Los Angeles [62–65]. Three researchers (DPK, JST, and KCO) independently reviewed transcripts and tagged text segments in Dedoose to identify, characterize, and categorize the key themes. The purpose of this review was to identify and classify key points that summarized the likes and dislikes of participants about the intervention session and if they considered it helpful in reducing AOD use and helping new residents get the support they need to reach their goals during the transition into housing. Using a grounded theory approach [66], key points with similar concepts were grouped together into a thematic category if mentioned by different participants. Text segments that illustrated themes were tagged using open coding by the three coders. Classic content analysis was used to identify quotes that fit each of the themes [67, 68]. Next, team members reviewed the entire list of tagged quote excerpts, identified coding disagreements, discussed reasoning behind coding decisions, and then came to a consensus on a final set of tagged quotes. Text segments were then exported into a spreadsheet and sorted by theme to identify sub-themes. Iterative discussion and sorting resulted in modifications of themes and sub-themes, including collapsing multiple sub-themes into one and expanding single sub-themes several sub-themes. A final summary description of each theme was written into a codebook.

#### Quantitative analyses

We produced descriptive measures of the participants demographics and satisfaction with the sessions. We also produced descriptive measures of the perceived usefulness of the sessions for new residents.

## Results

### Staff focus group and role play feedback

Staff who participated in the initial focus group reacted positively to the role-play demonstration of a session and provided suggestions for addressing some initial concerns. Staff made several comments expressing positive reactions to the use of visualizations to prompt discussion of the social networks of their clients. For example, “Everything was really easy to follow.” One participant commented, “I’m seeing this as a really great tool. Because we’re going to do it at the moment that they step in at the facility…They can realize that current people that they’re interacting with are not necessarily the most healthy for them…I’m excited about that.” Comments included discussion that the visualizations would be beneficial for interacting with YEH who at times have difficulty maintaining focus in usual case management: “just to be able to see it, just to be able to see it on paper, I think it’s more concrete. So, I think it would really help.” The group also discussed the benefit of having visualizations for case managers: “Especially having those graphs I think would be really great to help us out, to like show them, ‘This is what you’re telling me basically.’ So, I think that could really help us out…So being able to—like, at the end being able to summarize with that picture would help us out with our case management a lot more.” Staff also commented that the content of the session overlapped with their typical case management sessions and addressed their challenges mixing clients who are sober with those who are actively using: “these are things that we basically talk about with the clients.” The group confirmed that sessions would be possible to deliver every two weeks in case managers’ offices, which included privacy and computers that could access a link to a website. The group also expressed positive comments about the intervention’s potential to trigger changes in the social networks of their clients: “I think it’s like a spark that makes them think about it.”

The group also discussed concerns about some of the content of the sessions and offered suggestions for improvements, including changes to the text on screens and the visual displays. Group members identified screens that had difficult-to-read text or colors that were hard to distinguish and suggested ways to improve visibility of several screens. One comment emphasized the need for clear and straightforward wording: “You want to make sure that you provide as much detail as possible, especially if you’re going to ask information that is very personal to them.” The group expressed concern about clients who might not name 15 people or might be reluctant to name people who case managers might know. Discussion emphasized the usefulness of being able to skip ahead for those who can’t name 15 people and the usefulness of clients being able to use nicknames: “Just emphasizing that point that we're aware that they know that they can provide a nickname. It doesn’t have to be someone’s actual name…If they know, ‘Hey, you could provide nicknames’, then they’ll be more forthcoming at that point.”

### Resident post beta test satisfaction survey

On average, participants rated the session overall and each of the network diagrams as being between “Moderately” and “Very” helpful (overall mean = 2.5, sd = 1.5; network structure diagram mean = 2.5, sd = 1.4; network AOD use diagram mean = 2.7, sd = 1.4; social support diagram mean = 2.8, sd = 1.5). Participants rated the quality of the session overall as “Good” (mean = 3.3, sd = 0.7). On average, participants agreed with positive statements about the sessions overall (mean = 4.4, sd = 0.39) and with statements about how well the session would work for new residents (mean = 4.4, sd = 0.35).

### Resident post beta test discussion thematic results

Analysis of interviews conducted after beta test sessions revealed four broad categories of themes: (1) Acceptability, (2) Positive Benefits, (3) Visualization Reactions, and (4) MI-SNI interface reactions. For the Acceptability theme, three sub-themes emerged: (1) *understandability*, (2) *enjoyability and ease of use*, and (3) *barriers to acceptability*. Five sub-themes emerged for the Positive Benefits theme: (1) *learning/new insights*, (2) *enhancement to motivation to change*, (3) *making AOD changes*, (4) *building social support*, and (5) *usefulness to some but not others*. Five sub-themes also emerged from discussion of the visualizations: (1) *general positive comments*, (2) *understandability*, (3) *new insights*, (4) *triggering changes in social support*, and (5) *triggering changes in AOD use*. When discussing the MI-SNI interface beyond the visualizations, three thematic categories emerged: (1) *name listing*, (2) discussion of *screen wording*, and (3) discussions of the use of a *computer interface* to deliver the intervention. Each theme is described in detail below. Tables 1, 2, 3 and 4 provide additional and extended participant quotes illustrating each theme. Each table includes quotes organized by sub-themes, which are separated into rows, and identifies which comments were made by the same beta test participant with a code preceding each quote (e.g. [BT11], [BT12], etc.).

**Table 1:** Illustrative quotes for acceptability theme

Theme	Participant quotes
Understandability	[BT12]: “It all made sense” [BT13]: “It’s fairly straightforward and for a majority of people…Did a good job at making it clear.” [BT11]: “It went smoothly. It was easy to comprehend what was going on.”
Enjoyability and Ease of use	[BT32]: (Q: Why would you recommend it?) “Just for its simplicity: one, two, three. It won’t take you too long…I think that it's super simplistic, super straightforward…It doesn’t take long at all, so people that have anxiety and stuff can easily do this session…The length of it’s fine. That’s actually perfect for people with anxiety like myself. I don’t like really sitting down for too long and having to answer a lot of questions. So, this is perfect…I was just like, okay, this is going by actually pretty fast.” [BT14]: “I didn’t feel like it was long…It’s not that long… I thought it was really good, the length was good.” [BT14]: “It’d be interesting. Very interesting because it’s definitely going to change, at least for me it would change a lot…There’s weeks where I’m here a lot, weeks where I’m out a lot, like you’re at school or looking for a job…So there’s just a lot of people that I actually talk to…you said it was going to be four times? Well, if you do…that would helpful.” [BT14]: “It doesn’t only have to be about this. It can be about other stuff. It can be used for high school students…This is actually a very versatile thing.” [BT31]: “I enjoyed it. It was very relaxing. I didn't really feel stressed out. And I just had surgery a couple days ago…I really enjoyed this. This was very fun…I thought I was going to dread today… but I enjoyed it. I really had fun.” [BT12]: “I actually liked it.” [BT14]: “I actually liked this.” [BT32:] “It’s not really invasive, like most questionnaires would be [BT32]: “I think I liked everything.”
Barriers to acceptability	[BT14]: “For me, I can’t sit through things like that…so it’s helpful that somebody was right there trying to help doing it.” [BT14]: “Just how you’re saying it is like it would be when you do the two-week intervals, would be the best thing.” [BT32:] “It’s kind of weird, especially from the angle that I was doing it. She is my case manager, so it’s even weirder to peer over her shoulder. So, I was like, um…I don’t like doing this…if I was going to be forced to do this, I would have just said anything to get it over with.” [BT32:] “When I first moved in here, I don’t think I would have done this…Because before this I was homeless. I was not really in…anywhere, so I really didn’t communicate with anybody. Before that I was in a shelter, and even then I didn’t talk to anybody except the case manager to help me find housing. So I think it would be something that would be more appropriate if it was somebody who was here for maybe one month. Who they start making connections with here.” [BT13]: “I’m not really too keen on documenting all that. I’d rather do it on my own than…you know, be more private about it…I know you guys are confidential everything and all that, but it’s just the way that I feel.” [BT13]: “It’s something that’s pretty obvious that a lot of people do without even really realizing…it crosses people’s minds about how their relationships are and who they should interact with more, and who are the real supportive people and then who are the people that are just enablers and whatnot.” ([added]: “But nobody does it as thoroughly”) [BT11]: “Maybe offering a plan, like okay, what did you not like? Asking specific questions about the graphs or whatever and then being like what can you do, who can you go to to get what you need, or just coming up with a little plan for the next two weeks to refer to… because that was me coming up with…and my best thinking got me in a homeless shelter.”

**Table 2:** Illustrative quotes for positive benefits theme

Theme	Participant quotes
Learning/new insights	[BT12]: “It would maybe make you realize more.” [BT13]: “The possibility’s always there that it can make them more aware and change their life” [BT14]: “Just how everybody that I talk to is connected. It made me think about their relationship… And I was over here thinking I wonder what their relationship’s like?” [BT31]: “And it made me kind of learn that I actually have to go through with my goals.” [BT31]: “I learned who supports me and who I can depend on.” [BT12]: “Made me realize…my community that I reach out to and stay connected to.” [BT11]:“It doesn’t offer a solution. It just offers a note of self-awareness but it doesn’t offer any solutions. If they just have that information but no way to change it, then they’re not going to make a change.”
Enhancement of motivation to change	[BT31]: “I benefitted from it and I've been here for seven months and I want to change.” [BT32:] “You might learn something and you might change your outcome—your outcome might change your whole entire life.” [BT13]: “It can make them more aware and change their life” [BT12]: “It depends like your point of view, how motivated you are” [BT14]: (Q: would it be interesting to see what the change is?) “It’d be interesting. Very interesting because it’s definitely going to change, at least for me it would change a lot because I’m always doing different things. There’s weeks where I’m here a lot, weeks where I’m out a lot, like you’re at school or looking for a job…So there’s just a lot of people that I actually talk to.” [BT11]:[counter example]: “If they just have that information but no way to change it, then they’re not going to make a change.”
Making AOD changes	[BT31]: “Anybody who wants to make a change in their life and is willing to stop their substance abuse or anything, I feel like they can benefit from it.” [BT32:] “People that are reluctant to talk about their substance abuse would be more open to doing so in this format.” [BT11]: “Just thinking about who do I talk to on a daily basis. Are they supportive in my life? If I was in that mindset, would I use with these people and would they enable me to use with them? It was good to think about that.”
Building social support	[BT11]: “That might give them the motivation they need to get someone in their life that they can rely on…they can start creating a stable foundation for themselves if they’re unhappy with their support or lack of support in their life currently.” [BT13]: “I think it would help them adjust…Like how much you really interact with those people and whether or not they’re going to be helpful at the moment or if they’re going through it themselves, or if they’re an enabler, or just those people that are genuinely supportive and will continue to be supportive.” [BT31]: “I wish—like there's a boy… who used to be here. He had a substance abuse problem, a drinking problem, and he just got exited. I wish that this would have been there when he was here, because I know he personally could have benefited from this and I know that he needs it… he has a problem and he needed help and this probably would have helped. And it would have made him realize everyone who supports him and you know, how to cut people out and it would have—he would have benefitted so much from it. And I just wish he could have did it.”
Usefulness to some but not others	[BT12]: “It depends like your point of view, how motivated you are.” [BT13]: “For me personally, it’s not really helpful for me as an individual...I got my own whole plan that I don’t necessarily share with anybody…for somebody else that hasn’t really thought about it too much and is not as aware as I am, I think it would be a lot more helpful for them. I see that it can do a lot of good and really help other people…It depends on who they are, the little bit of information that they divulge, I guess.” [BT13]: “But there’s a lot of people that I know that could really benefit from this type of thing because it really puts it out there how these relationships with certain people affect everything.” [BT13]: “If you’re willing to really take into consideration everything that is given to you clearly on the diagrams and all that, and makes you want to change your environment; then yeah, I think it would really help.” [BT13]: “The possibility’s always there that it can make them more aware and change their life, I guess.” [BT13]: “For a majority of people that come through here, I think it would be helpful for some. For—yeah, for most, actually.” [BT14]: “It would be good for somebody who’s been here a while.” [BT31]: “Maybe for other people, like I'm pretty advanced and I'm pretty smart. So, I get things more than an average person does. So, I can't judge. I don't really know a lot of these people here. So, maybe you could do it like more intuitive for people who don't understand but also not make it too complicated.” [BT31]: “Yeah, I mean, if anybody is willing to make a change in their life, it's beneficial for them…But they have to want to make a change…Because you can't save somebody who doesn't want to be saved.” [BT32]: “It’s really depending upon the person’s struggle.” [BT32]: “It’s going to be like a challenge for those people that are trying to change their patterns, like change their life. Because if they have to name people that they don’t even associate with and they see that result, it’s like…this means nothing then.”

**Table 3:** Illustrative quotes for visualization reactions theme

Theme	Participant quotes
General positive comments	[BT14]: “Just seeing the different graphs” [BT11]: “I liked the visualization of the support system.” [BT32]: “I enjoyed seeing the diagrams. It’s like the first time that I’ve seen any type of diagram system, so it was nice.”
Understandability	[BT11]: “I think it was helpful in the way that it kind of validated like I’m not alone and I do have a lot of people that I can depend on that…I don’t just have people in my life that I talk to constantly that aren’t there for me. I have people in my life I talk to that care about me. So that’s good to validate that.” [BT12]: “It shows you the people that are connected with each other and know each other…the fact that it gets you out of your head and it’s more organized. It’s on paper and you can look at it a little more clearly. That’s a big plus” [BT13]: “It made sense to me but it’s not really helpful…I believe everybody’s different, so a majority of people are visual learners. I believe it will help the majority of the people that come through.” [BT31]: “I feel like the ones who support you are important, but I feel like the ones who are not supportive of you are important, as well; that should stick out more because you need to understand, like, why—if those people you can't be supported by them, then you have to realize within yourself why you need to let them go, if they're not going to support you—like how I said, if it's not beneficial for you, you shouldn't really have it…Maybe—because red is a very stick-out color, so red—I like that for support, but you also need to find another color that's bright and brings your eye up, to make you realize these people don't support you.”
New insights	[BT11]: “Seeing the interconnectedness of my interpersonal relationships, that was interesting…I have good groups of people in my life that are different but are equally reliable.” [BT12]: “You can figure out your connections and your…the kind of people that you’re connected and trusted.” [BT12]: “Being able to compare the relationships, not just with you and those people that you list, but with those people between them, too and how each person can affect you…helps you compare how you interact with certain people versus the people that are more supportive and whatnot.” [BT14]: “I was able to actually see my relationships and how they are built, whether I can talk to people, whether I hang out with them and do substances and drinking with them.” [BT14]: “Didn’t really think about that because I talk to these people every day, and then I saw it and I’m like I didn’t realize that they knew…that I’ve introduced them and they’ve talked before.” [BT31]: “I liked the graphs…to make me realize and understand I have these people who support me, even though just saying it, seeing it is a whole different thing and knowing these are the people I have support and that they support me.” [BT32]: “The showing of like your support groups and the people that may have an impact on you if they’re users and whatnot. That way you can see really—like actually put into visual who’s not helping you anymore and why your life is the way it is…It was just these are your circles and this is where the lines go. They might be chaotic but that’s because your life is, well, this is based off you, you know?” [BT32]: “The diagram showed how many people are not there and how many people are maybe there.”
Triggering changes in social support	[BT11]: “That might give them the motivation they need to get someone in their life that they can rely on.” [BT11]: “I could use that as a tool to reevaluate some relationships I have in my life.” [BT12]: “I learned the difference between the people that are connected and motivated. And we reach out to each other…And I probably…I noticed the people that I wouldn’t hang out with, you know?” [BT14]: “At the end you can see who’s been the constant, right there. And then decide…. you can see what you’re doing and how you can change it, who you’re hanging out with, who’s been somebody that you’re always in communication with.” [BT14]: “It kind of made me realize that I need to build more connections…when I saw it was all connected it helped me because I was like I need to build better connections, more personal connections…Because if you do, either when they leave or you leave, you can keep in contact and you can just have somebody there always like that, who understands where you’re coming from and just helps you out.” [BT32]: “The people that are outside sources are labeled even in the visuals as outside sources…that way you can see like if you need help, you can reach out to these outside sources.” [BT32]: (Q: Do you think seeing the diagrams will help people turn to support more?) “I would imagine so. That was my thought process when I saw it all…as I was seeing it visually, I was like, yeah, I would turn to these people more often than these other people.” [BT32]: (Q: Do you think seeing the diagrams will help people change their social networks?) “If they’re openminded with their representation of what works and what doesn’t, you know, just based off their diagram alone might help them redesign their social networking structure. Maybe.”
Triggering changes in AOD use	[BT12]: (Q: Do you think seeing the diagrams will help residents change their drinking?): “Because (they) pretty much…guide you… Showed you…the people that you don’t have healthy relationships with.” [BT14]: “It shows you who you’re most likely to drink with versus who you’re most likely not to, so you’re going to be like, okay, I’m not going to hang out with person, he’s having drinks. So limit or set boundaries for myself and for them.” [BT32]: (Q: Do you think seeing the diagrams will help residents change their drinking?): “Possibly…Because the way that it’s formatted makes you really think about things. But at the same time, if the person isn’t really willing and does the session, it could go either way.”

**Table 4:** Illustrative quotes for MI-SNI interface reactions theme

Theme	Participant quotes
Name listing	(Q: was it difficult naming 15 people?) [BT11]: “Kind of hard.” [BT12]: “Not at all…Fifteen; I think that’s a good amount of people.” [BT13]: “Moderately, just on the spot…by the end of the 15 names I thought of another whole different set of names that I could have said and changed the outcome of the graphs and all that.” [BT14]: “It was a lot of people to really think about. I was like okay, 15 people. I was kind of reaching for people that I actually talk to…I feel like it’s too many…Ten to 12, maybe. Ten or 11. Ten, 11, 12, one of those because… for me it’s like I’m homeless, I do use some substances and I don’t like to have a big circle. I have little, close people that may know what I do or I do it with them, I get it from them and stuff like that. That’s for me. Because some of those people I talk to only in here, not outside of here…I wish I would have brought my cell phone so I could see who I’ve been talking to.” [BT31]: “That actually wasn't very hard.” [BT32]: “Some people such as myself, we don’t have 15 people to name off. Sometimes we only have like three people in our lives…(Q: But you were able to come up with 15?) They just weren’t all close buddies…I don’t really talk to people so it’s like this is hard. So that comes up, it’s going to be like a challenge for those people that are trying to change their patterns, like change their life. Because if they have to name people that they don’t even associate with and they see that result, it’s like…this means nothing then…Like incorporate even it’s just somebody that you say hello to once in a while, whether it’s a good person or a bad person. Just notate it like if you don’t have many people in your life, basically, include any outside sources.” [BT14]: (Q: was it comfortable providing names?) “The people that I named I know wouldn’t mind if I talked about them like that… if I come up with nicknames and stuff, later in time I’m going to be like, who the hell is this?…If I gave initials, I’m like, oh, this is going to be this person and this person, but who was I talking about?”
Discussions of screen wording	[BT11]: “The one part where it says people that you would use with and it says use a normal amount and then use more than—I think I’m a real addict, so there is no normal use for me. When I use, it’s excessive and there’s no moderation and there’s no control. So I think when non-addicts take it, they can relate to that and be like oh, yeah, I didn’t really want to get that fucked up. But if a real addict like me is taking it, every time with everyone if I ever use with them, it would be excessive. Every time.” [BT14]: “I liked how they asked about, what are your immediate goals, what do you want to accomplish?” [BT31]: “I also liked the way that the questions were worded and how you worded them to get the point across and also it helped me understand more better.. I probably wouldn't have said the right thing or what I actually meant, if the questions were worded differently…Also the choices that you had were very good choices. Do you know what I'm talking about? Like when you pick the two things, they were good choices, too.” [BT31]: “I would like to pick more than just two places to improve, because I wanted to pick all of them, because I feel like I need all of them. I need to improve my mental health and substance abuse and get out of here and keep my job and—you know? So if there could be more options.” [BT31]: “And then I noticed with the substance use, there wasn't an option for “using less with”…Because I don't want to be using the same amount. I want to slowly go off, to where eventually I'm at a point where I don't need it anymore. I don't have to do it. I've found different coping skills, I've found another way, you know?…Because I don't want to use more, I don't want to use the same. I want to slowly use less.” [BT31]: “It felt very scripted and very monotone and not flowing…I would rather have it to where she just says what rolls off right and it doesn't feel like she's being, say this and this and—you know?”
Computer interface	[BT13]: “I guess nowadays it’d be a lot easier doing it on a computer. But I think that they should get a case manager or somebody else to be there with them and bring up new ideas that they’re not necessarily thinking of at the moment.” [BT32]: “Sometimes the pages become extremely too bright. So in technical format, dull it down. Dull the brightness down.”

### Acceptability

Table 1 provides detailed quotes that illustrate the Acceptability theme. Discussions about the experience of participating in an intervention session included comments from participants about their own positive and negative experiences as well as how acceptable they thought it would be for other participants. Participants described reactions to their experience as well as suggestions for improvement. In general, participants positively characterized the session’s *understandability* through comments about how the session made sense and was simple and straightforward. Participants also described the intervention’s *enjoyability and ease of use* by stating various comments about how much they enjoyed participating in the session*.* Some comments emphasized that this positive experience was a surprise to them.

In debrief sessions, there was some discussion about potential *barriers to acceptability* and how they could be addressed. One participant remarked that he would not normally be able to sit through a session like the MI-SNI session, but added that doing a session with a case manager made it possible. He also expressed approval of the plan to schedule four sessions conducted at two-week intervals because that would provide enough time to see how his social network changed. Another participant remarked that having to look over the case manager’s shoulder to see the MI-SNI screens made him uncomfortable. He also added that it would be better to start sessions with residents after they have been living at the housing facility for at least a month, because discussion of connections would have been difficult right away. Another participant remarked that the session questions made him uncomfortable because he preferred to be private. He also added that he thought the topics covered were things that people already considered naturally, but added that they were covered more in depth in the MI-SNI session. Another participant mentioned that the session itself would not be sufficient to help residents who have histories of making bad choices and that it would have to be combined with a plan on how to deal with issues that were discussed.

### Positive benefits

Table 2 provides quotes illustrating the Positive Benefits theme. Participants discussed the potential of the intervention process for producing positive benefits for participants. Most of the participants discussed how the intervention triggered *learning or new insights* and facilitated greater awareness and learning about their lives and relationships. Other participants provided additional details about how the session triggered learning. For example, several participants stated that they gained insight into their social relationships, including who supports them with achieving goals.

Most participants discussed how these insights would help establish a foundation for making changes by *enhancing motivation to change* and that these changes could have a significant impact on a participant’s life. One participant commented that he would be very interested in seeing how the sessions triggered change over time. In contrast with participants who linked learning during sessions with enhancement in motivation to change, one participant commented that learning alone would not accomplish changes because learning needed to be paired with guidance on how to solve problems and make changes.

When discussing how the MI-SNI session would help residents make changes, discussion focused specifically on the usefulness of the session for making *AOD use changes* and *building social support*. Some stated that MI-SNI sessions would help reduce AOD use and enable discussions with those who might be reluctant to talk about AOD use. Participants stated that they felt these discussions would trigger motivation to add supportive network members and raise awareness of who in the network might enable AOD use. Other participants noted that the beneficial effect would help new residents in particular evaluate and build social support. One participant remembered a former resident with AOD use problems who left the program and thought that someone like him would have benefitted from receiving MI-SNI sessions. While participants discussed the benefits of the MI-SNI for triggering change and enhancing motivation to change, they also expressed some qualifications for when the tool would be useful and when it would not help trigger change and emphasized that the intervention would be *useful to some but not others*. Willingness and prior motivation to change were cited by participants as a necessary factor for the MI-SNI to lead to changes. One participant stated clearly that he did not think the tool would help him, because he had already planned out his approach to making changes, but he did consider it a potential beneficial tool for others. Another participant suggested that, although she understood the session, it should not be too complicated because some residents might not understand it.

### Visualization reactions

Table 3 provides quotes that illustrate the Visualization Reactions theme. When asked what they most liked about the session, many participants made *general positive comments* about seeing the visualizations of their networks and identified that as the most enjoyable part of the intervention experience. Participants generally responded affirmatively when asked if the graphs made sense indicating high *understandability* of the visualizations. One participant stated that this validated a sense of network support and another stated that seeing the graphs helped make thoughts about network connections clearer than simply thinking about them. One participant who said that the graphs made sense to him added that he did not find them useful because he was not a visual learner, but thought they would be helpful to most other residents. Another participant thought that the diagram depicting support made sense, but suggested that those who do not provide support should also be emphasized.

In addition to affirming that the graphs made sense, many of the participants comments mentioned *new insights* they gained from looking at the diagrams, such as noticing the types of people who they could rely on and which network members were connected to each other. One participant stated that he was better able to understand dynamics within his relationships and how this related to substance use. Another participant said that viewing a diagram of a network provided more insight about support in her network than merely discussing social relationships.

Several participants mentioned that viewing diagrams would help trigger improvements in their social networks including *triggering changes in social support* and *triggering changes in AOD use*. Participants mentioned that seeing diagrams would help motivate them to make positive changes to their networks and re-evaluate relationships, including adding people to their networks who would increase support or avoiding other people, in particular those who influence AOD use.

### MI-SNI interface reactions 

Participants also commented on what they liked and did not like about the M-SNI interface beyond the network diagram displays. When asked about their experience of *name listing*, specifically how difficult it was to name 15 network members that they recently interacted with, participants gave a range of responses, from difficult to not at all difficult. One participant who described some difficulty was able to name 15 people and stated that it was possible to keep naming more after the initial 15. One suggested that checking call records on a cell phone would help trigger memories of interactions. One participant who provided 15 people said that the list included those who were not very close and recommended instructing future participants that they do not have to name only strong ties. None of the participants expressed concerns about naming people they knew in the session. One participant cautioned against using initials or nicknames instead of familiar names to make the process more anonymous because it would make answering subsequent questions about those people more difficult.

Participants were generally satisfied with the wording of the MI-SNI interface questions with some minor suggestions and did not express much concern about the session being delivered through a computer interface. Some commented that they liked the wording. One participant commented that case managers should not read screens word for word and the interaction should be more free-flowing and conversational. Some offered suggested changes, such as listing more than 2 options for goals to discuss in the session. When discussing questions about the patterns of use with network members, one participant suggested not referring to a “normal” amount because it would not be relevant to those who used drugs and alcohol at a high level consistently. Another participant suggested adding an option for using “less” than usual with those who were helping them cut down on their use.

## Discussion

For YEH transitioning into supportive housing, this period provides a window of opportunity to make positive changes in their social networks that can have important implications for future AOD use, housing stability, and goal achievement. In the current study, we tested the acceptability of a motivational interviewing-based social network intervention for YEH that aims to focus on AOD use and social network changes. Consistent with our previous study with adults transitioning into supportive housing, we found the intervention to be acceptable, useful, and enjoyable to program staff and YEH residents. Our approach to adapting the intervention is consistent with findings from recent studies that emphasizes the diversity of types of support and social capital available to YEH [13–15, 17]. Our intervention approach provides a tool for case managers to assist YEH in identifying and activating these supports to help them build resilience and overcome individual vulnerabilities as they support their transition away from homelessness. Our approach is also consistent with conclusions from recent studies that emphasize the need for the development and testing of social network interventions to prevent and decrease AOD use among YEH [12]. The findings presented here give support to an approach that combines motivational interviewing and visualizations of personal networks in addressing both social networks and AOD use as YEH transition away from living on the streets.

The discussions with supportive housing staff and residents about the proposed intervention provide preliminary evidence that the adaptation of the MI-SNI for YEH was successful. Staff who participated in demonstration discussions or beta tests commented that key elements of the intervention, such as the motivational interviewing approach, the focus on goals, and social networks corresponded with their usual approach to case management. They also commented on the expected usefulness of social network visualizations for engaging with YEH. Two of the beta tests described here were conducted by supportive housing case managers, who successfully used the MI-SNI interface to ask questions and enter the responses that were used to produce network visualizations. This represents an advancement over the previous use of the MI-SNI with adults experiencing homelessness in which sessions were facilitated by research staff. The use of a computer interface to deliver the MI-SNI was not considered a challenge by the case managers, who were able to log into the system website using their own staff computer equipment, which did not require installation of additional software. The interface offered flexible options for delivery of case management sessions to residents, including usual case management sessions in a case manager’s office as well as virtual sessions using video conferencing and screen sharing technology when residents were unable to attend sessions in person. The ability of case managers to deliver the MI-SNI using their usual office setting and equipment demonstrated the feasibility of housing staff to deliver the MI-SNI directly to their residents.

The reactions of residents who took part in MI-SNI beta test sessions also provide evidence of the success of the adaptation. Residents expressed many comments confirming that they understood the information they were provided and that they enjoyed the experience of participating. They also discussed a range of potential benefits of the session for other residents, including benefits for building supportive connections and reducing AOD use. Participants provided many positive comments about the network visualizations they were shown and stressed the key role they could play in the MI-SNI sessions’ goal of helping a resident make positive changes in their lives.

Although most of the comments were positive, there were some concerns and criticisms provided by staff and beta test participants. Many of these comments provided an opportunity to improve the intervention design and the MI-SNI interface. Some of the comments also provided valuable information for the development of a case manager manual and training procedures. For example, the colors used in the network visualization were adjusted to improve distinguishability among types of nodes. Also, case managers were instructed on how to interpret highly dense networks with many connections among network members by emphasizing the node clustering rather than trying to understand each individual line connecting nodes. One concern expressed by staff was the challenge of naming 15 people and the concern that some residents might have for naming specific other people. When asked about how difficult it was to name 15 people, beta test participants described it as somewhat difficult at first, but ultimately a task they could complete. Based on the feedback we received, we did not adjust the overall number of names to elicit, but we did program the name entry page to allow a case manager to skip ahead if a participant was unable to provide all 15 names. We also provided instructions to case managers to prompt residents to name people who were not necessarily their closest or best friends and that they could name anyone who they had a recent interaction with, even if this was not face-to-face. In addition, we provided instructions to case managers that they should emphasize that if they did not feel comfortable providing names that they could also provide initials, nicknames, or descriptions as long as they would be able to identify who each of the network members were later.

Other comments regarding wording of MI-SNI interface questions identified issues related to AOD use and identifying session goals. At the recommendation of staff, we added a question to capture the participant’s primary drug of choice and the displayed this substance on the screen with the AOD network visualization and included prompts to focus on this substance when discussing network influence. We also modified the wording of the question that prompted residents to classify their AOD use with network members. One resident mentioned that classifying the amount of AOD use with network members as “normal” would not apply to those who had severe AOD use problems. We adjusted the wording to “regular” to emphasize deviations from their own personal pattern of use triggered by their interaction with a network member. We also modified this option to include a reference to using less than the regular amount with the network member to capture relationships where the resident and a network member were taking steps to reduce use together. We modified the goal selection question based on feedback from staff focus group discussions to include specific references to physical and mental health rather than referring to both together as “health”. We also considered expanding the number of goals that a resident could select as a session focus based on a comment from a beta test participant. We continued limiting the number of selected goals to two after staff emphasized that it was important to simplify and focus case management sessions. To enable a broader discussion of goals, we added programming to allow the selection of new goals during each session, which provided residents the opportunity to discuss more than two goals over the course of their four sessions.

The results presented here represent the first phase of a clinical trial development and pilot test grant. These results provide empirical evidence of the acceptability of the intervention approach and lay the groundwork for a pilot test with a similar population of YEH transitioning into supportive housing. This process of beta testing prototypes of an intervention approach is consistent with expert guidance on behavioral therapy research [69]. Further stages of development will also follow these guidelines with the completion of a small pilot study of YEH transitioning into supportive housing with random assignment of either intervention delivery or usual case management to explore the efficacy of the intervention approach. Recruitment and enrollment in this pilot study is ongoing [70] and will inform a larger clinical trial if preliminary evidence of the intervention’s efficacy is established.

### Limitations

Although the results presented here are promising, the current study does have some limitations. The small purposive sample of staff and residents who provided feedback is appropriate for an initial beta test to gauge the experience of those who will use the technology and procedures in subsequent tests. However, this design limits the generalizability of the findings to other groups and these findings do not provide any indication of how effective the intervention will be when delivered to a larger sample. Although the sample size was small, the pattern of responses was fairly consistent across beta test sessions and additional beta tests would have been unlikely to inform additional modifications of the MI-SNI interface or procedures. Also, we only conducted beta tests with participants for one session. Although the structure of the sessions is identical, we did not assess the experience of participants receiving more than one session. It is possible that there are areas of improvement that we were unable to identify with the limited number of beta test participants and sessions. However, the beta testing results presented here met our goals of adapting the initial MI-SNI for YEH transitioning from homelessness to supportive housing and for delivery by their case managers in preparation for a pilot study. The exclusion of any explicitly negative questions among our set of satisfaction questions is another limitation. We did include response options that ranged from negative to positive for five neutral questions about overall satisfaction and helpfulness, but the eleven agreement questions were all phrased positively. We believe that this limitation is mitigated by the mixed-methods interview design in which closed-ended structured questions were followed by open-ended questions that enabled participants to express, in their own words, what they liked and did not like about the session. The themes that emerged from analyses of these open-ended responses did not deviate substantially from the range of responses to the closed-ended questions.

## Conclusion

This study is the first adaptation of an innovative “network alteration” behavior change intervention. In addition to the adaption for delivery to YEH who have experienced homelessness, the work described here represents the first adaptation of the MI-SNI for delivery by case managers to clients. The intervention was adapted with iterative feedback from supportive housing staff and current residents of a housing program that focuses on providing services to YEH. The purpose of this adaption is to reduce AOD and increase/strengthen their stable, prosocial supportive connections among YEH transitioning out of homelessness. Previous research suggests that this transition may serve as a critical time to intervene to prevent future risk and improve outcomes. Our results show that the intervention was perceived as acceptable by staff and residents. More research is needed with a larger sample and longer time frame to explore the potential effectiveness of the intervention in reducing AOD behaviors and building social support.

## Data Availability

Once collected, deidentified data from this study will be available from the corresponding author on reasonable request one year after all aims of the project are completed. Requestors of data will be asked to complete a data-sharing agreement that provides for (1) a commitment to using the data only for research purposes and not to identify any individual participant; (2) a commitment to securing the data using appropriate computer technology; and (3) a commitment to destroying or returning the data after analyses are completed.

## Abbreviations

SUD: Substance use disorders
YEH: Young people experiencing homelessness
AOD: Alcohol and/or other drug
SNA: Social network analysis
MI: Motivational interviewing
MI-SNI: Motivational Interviewing social network intervention (MI-SNI)
